# Machine learning in neuroimaging: from research to clinical practice

**DOI:** 10.1007/s00117-022-01051-1

**Published:** 2022-08-31

**Authors:** Karl-Heinz Nenning, Georg Langs

**Affiliations:** 1 Center for Biomedical Imaging and Neuromodulation, Nathan Kline Institute, Orangeburg, NY USA; 2 Department of Biomedical Imaging and Image-guided Therapy, Computational Imaging Research Lab, Medical University of Vienna, Währinger Gürtel 18–20, 1090 Vienna, Austria

**Keywords:** Neuro imaging, Artificial intelligence, Deep learning, Connectomics, Prediction models, Neurobildgebung, Künstliche Intelligenz, Deep-Learning-Verfahren, Konnektomik, Vorhersagemodelle

## Abstract

Neuroimaging is critical in clinical care and research, enabling us to investigate the brain in health and disease. There is a complex link between the brain’s morphological structure, physiological architecture, and the corresponding imaging characteristics. The shape, function, and relationships between various brain areas change during development and throughout life, disease, and recovery. Like few other areas, neuroimaging benefits from advanced analysis techniques to fully exploit imaging data for studying the brain and its function. Recently, machine learning has started to contribute (a) to anatomical measurements, detection, segmentation, and quantification of lesions and disease patterns, (b) to the rapid identification of acute conditions such as stroke, or (c) to the tracking of imaging changes over time. As our ability to image and analyze the brain advances, so does our understanding of its intricate relationships and their role in therapeutic decision-making. Here, we review the current state of the art in using machine learning techniques to exploit neuroimaging data for clinical care and research, providing an overview of clinical applications and their contribution to fundamental computational neuroscience.

## Abstract (de)

Die Neurobildgebung ist bei der klinischen Behandlung und Forschung ein entscheidender Faktor, und ermöglicht es, das Gehirn in gesundem und krankem Zustand zu untersuchen. Es besteht ein komplexer Zusammenhang zwischen der morphologischen Struktur, der physiologischen Architektur und den entsprechenden Bildmerkmalen. Die Form, Funktion und Verbindungen zwischen verschiedenen Hirnarealen verändern sich während der frühen Entwicklung und im Laufe des gesamten Lebens, sowie bei Krankheit und Genesung. Wie nur wenige andere Bereiche zieht die Neurobildgebung einen Nutzen aus fortgeschrittenen Auswertungsverfahren, mit denen Bildgebungsdaten analysiert werden, um das Gehirn und seine Funktion zu untersuchen. Seit Kurzem leistet auch das maschinelle Lernen ein Beitrag (a) zu anatomischen Messungen, der Erkennung, Segmentierung und Quantifizierung von Läsionen und Krankheitsmustern, (b) zur schnellen Identifizierung akuter Erkrankungen wie zum Beispiel Schlaganfall oder (c) zur Nachverfolgung von Veränderungen über die Zeit. Mit den Fortschritten bei der Neurobildgebung und ihrer Auswertung nimmt auch das Verständnis der komplexen Beziehungen zwischen Struktur und Funktion und ihrer Bedeutung für die therapeutische Entscheidungsfindung zu. Für die vorliegende Arbeit wurde der aktuelle Wissensstand zum Einsatz von Verfahren des maschinellen Lernens bei der Auswertung von Daten aus der Neurobildgebung für klinische Behandlung und Forschung erhoben; klinische Anwendungen sowie ihr Beitrag zur als Grundlage dienenden computergestützten Neurowissenschaft werden im Überblick dargestellt.

## Background

Since recognizing the brain’s significance for human cognition, scientists have studied the intricate relationship between body and mind [1]. The complexity of the human brain has fostered an array of advanced neuroimaging techniques to quantify its structure and function [2]. These techniques provide insights for neuroscientific research, clinical evaluation, and treatment decisions.

Machine learning can identify patterns and relationships of signals in neuroimaging data. Classification and regression techniques detect and quantify clinically relevant findings with increasing reliability and accuracy. Yet, these models can do more than repeat what we train them to do. What if instead of trusting the neuroanatomy to guide the comparison of individual brains, we use their individual functional interaction structure itself to establish correspondences? Can machine learning help to create cortical maps of functional roles, or the influence of genes and environment? Machine learning may offer a tool to fundamentally change our perspective on the observations we make.

A tangible visualization of the brain’s anatomy and neurophysiological properties is essential for cognitive neuroscience and clinical applications [2]. Neuroimaging can be broadly characterized by two categories: structural and functional imaging. *Structural neuroimaging* aims to visualize the anatomy of the central nervous system and to identify and describe structural anomalies associated with traumatic brain injury, stroke, or neurological diseases such as epilepsy or cancer [1]. Structural MRI can also serve as a predictor for various neurological and psychiatric disorders [3]. *Functional neuroimaging *on the other hand captures the brain’s neurophysiological or metabolic processes. In clinical practice, it is important to understand neurological impairment and neuropsychiatric disorders, and to inform surgical treatment to keep vital cognitive function such as language or motor capabilities unharmed [4]. Although functional MRI (fMRI) finds broad applications in research, it is typically limited to task-based preoperative mapping of essential functions such as motor control in clinical routine [5]. Resting-state fMRI has emerged as a promising tool [6, 7] since it does not rely on the patient’s ability to perform a specific task. Although promising, the translation into clinical practice is still challenging [8]. Nevertheless, the importance of the interconnectedness of the brain has been acknowledged in the clinical context [9]. While fMRI is a central modality in functional imaging [10], other imaging techniques such as positron emission tomography (PET; [11]) or MR spectroscopy [12] can capture underlying metabolic processes and thus complement structural imaging. Characterizing metabolic properties in the brain is useful for a variety of clinical applications, such as defining infiltration zones and tumor properties in neuro-oncology [13, 14], understanding the pathogenesis of progressive diseases such as multiple sclerosis [15], or understanding neuroinflammation and psychiatric disorders [16]. Compared to structural imaging, functional neuroimaging can detect underlying neurophysiological and molecular properties, and combining both enables a multi-modal description of the brain and the modeling of its structure–function relationship as a marker for disease [17–19].

Computational analysis has long been part of neuroimaging, as the recorded signals must be translated into quantitative comparable measurements for which relationship to disease and treatment response can be assessed. The tool box includes techniques such as image-based registration to establish correspondence across individuals for summary and comparison of measurements [20], voxel-based morphometry (VBM; [21]) to quantify phenomena such as gray matter atrophy [22], or statistical parametric maps obtained by general linear models (GLM; [23]).

In this context, machine learning has gained significant importance. Early work advanced functional neuroimage analysis from univariate inspection of individual voxels as in GLM analysis [24] to localized activity patterns [25] and the detection of multivariate widely distributed functional response patterns [26, 27]. These distributed functional patterns offered the opportunity to treat and align their relationship architecture similarly to anatomical maps [28]. Decoupling the analysis of function from its anatomical location altogether enabled the assessment of instances where anatomy is affected by disease such as in brain tumors and reorganizational processes [29]. Machine learning led to a substantial acceleration in brain segmentation [30], image registration, and the mapping of individuals to atlases [31]. Finally, it has led to the ability to automatically detect and segment brain lesions such as tumors with high accuracy and reliability [32].

Here, we review the current state of the art of machine learning in neuroimaging. We structure the overview into three areas. The review of machine learning for structural neuroimaging includes the registration, segmentation, and quantification of anatomy as well as the detection and analysis of findings associated with disease. The overview of machine learning for functional neuroimaging encompasses multivariate analysis of response and the independent representation of function and anatomy as they may be decoupled through development or disease. Finally, network analysis describes how brain networks can be quantitatively assessed and represented. In each section we address research and clinically relevant applications of machine learning techniques. Finally, we summarize open challenges that may be tackled with machine learning approaches.

## Machine learning in structural neuroimaging

**Fig. 1:**
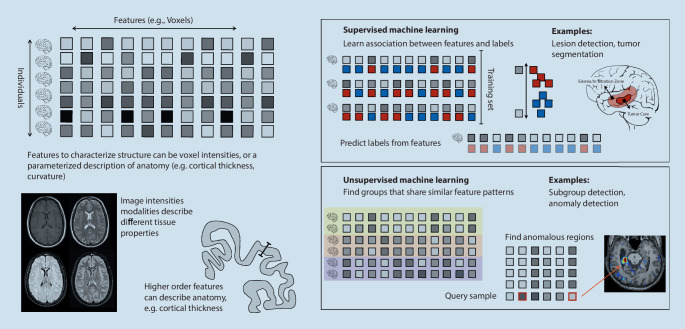
Machine learning can detect, segment, and quantify characteristics of anatomical structures and abnormalities associated with disease. Supervised machine learning learns from paired training examples such as imaging data and annotated tumors. Unsupervised machine learning identifies structures in a large set of observations, establishing normal variability or detecting groups of similar observations

Structural neuroimaging is the mainstay in clinical diagnostic neuroradiology. Although it can only capture overt structural properties of the brain, it is of great value in supporting the diagnosis and treatment decisions for various diseases [1]. It facilitates quantifying the size of brain structures and their deviation associated with disease as potential markers for clinical outcome [33]. Given the availability of structural neuroimaging, machine learning approaches aim to utilize the increasing number of available images to establish robust models for segmentation, classification, or prediction tasks ([3, 34]; Fig. 1).

### Structural imaging for quantification of anatomical structures

The accurate segmentation of neuroanatomical structures is important as a basis for their quantification and as a prerequisite for the analysis of possible anomalies of specific areas related to clinical findings, disease, and treatment response. While early work on brain segmentation focused on atlas-based tissue probability maps [35, 36], recent machine learning-based approaches aim to predict accurate segmentation labels. Deep convolutional neural network approaches were able to improve the segmentation of neuroanatomy compared to standard tools in both speed and accuracy [30]. Deep learning-based approaches have shown promising results for challenging applications of brain tissue segmentation, such as in neonates [37] and fetuses [38]. Furthermore, a U-net deep learning model has demonstrated the feasibility of highly accurate segmentation of neuroanatomical structures from CT scans [39]. The robust quantification of anatomical structures is important to establish markers of disease progression and outcome, and the introduction of deep learning-based U‑net models [40] has advanced the accuracy of medical image segmentation overall. Such models have been successfully applied to automatically quantify possible structural markers with prognostic values, such as temporalis muscle mass [41], white matter hyperintensities [42], or brain vessel status [43].

### Structural imaging for disease assessment

One important application of structural neuroimaging in clinical routine is lesion detection and characterization. The characterization of brain tumors benefits from the wide range of available neuroimaging techniques and modalities. A multitude of machine learning approaches have been applied to brain tumors to improve their delineation and characterization, informing treatment decisions to ultimately improve patient outcome. Deep learning approaches have improved lesion detection by quantifying anomalies in a model of normal brain structure [44]. Machine learning for fully automated quantitative tumor characterization contributed to the basis for clinical decision-making [45]. This potential benefit in neuro-oncology has also prompted scientific initiatives such as the Brain Tumor Segmentation Challenge (BraTS; [32]), highlighting the value of data sharing for methodological improvements. This challenge leverages multimodal MRI data from multiple institutions, bringing together the scientific community to evaluate and advance segmentation, prediction, and classification tasks within the highly heterogeneous brain tumor landscape. In addition to raw imaging data, this initiative also provides preprocessed data and radiomic features in an accessible open dataset to lower the barrier for the development of new machine learning approaches [46].

Radiomics has become a widely adopted approach in medical image analysis [47], aiming to describe lesions via tissue properties based on shape, intensity, and texture features [48]. Such features build the basis for unsupervised learning, aimed at identifying and characterizing subgroups based on their radiomic features, or classification tasks, assessing features for their discriminative power (Fig. 1). In the clinical context, machine learning approaches based on radiomic features have been used to identify subgroups of tumor patients [49] or to differentiate between primary central nervous lymphoma and atypical glioblastoma [50, 51].

Another area in which structural neuroimaging and machine learning are relevant is the assessment of patients with epilepsy. Here, the detection of often subtle cortical malformations or lesions is critical for informing treatment decisions. Supervised machine learning approaches such as artificial neural networks have been shown to identify focal cortical dysplasia [52, 53]. Unsupervised approaches including clustering techniques were able to reveal a structural anomaly landscape that defines distinct subgroups of patients with epilepsy [54]. A similar application of unsupervised machine learning was able to detect subtypes in multiple sclerosis that exhibited distinct treatment responses [55]. In acute stroke, where rapid treatment decisions are essential, machine learning has the potential to improve patient outcome by detecting the type of arterial occlusion or hemorrhage and informing short- and long-term prognosis [56]. Machine learning has also facilitated the linking of disease-related imaging phenotypes to their underlying biological processes. For instance, so-called radiogenomics in glioma patients showed promise to inform treatment decisions in a personalized medicine approach to support optimal treatment decisions [57].

## Machine learning in functional neuroimaging

Functional neuroimaging aims at capturing neurophysiological processes in the brain. The use of fMRI has given rise to mapping the location of cognitive functions across the cortex, so-called functional localization. This is relevant in basic neuroscience and in clinical applications such as the presurgical localization of eloquent areas. The first functional localization techniques treated each brain region independently in a univariate fashion [23]. However, as fMRI can image the entire brain, it enables the analysis of relationships between brain regions. Therefore, multivariate machine learning approaches, relating observations and relationships across multiple brain regions, are relevant [26].

### Detecting multivariate functional response patterns: encoding and decoding

The multivariate nature of the brain motivated machine learning approaches for functional mapping, aiming to map the anatomical location of cognitive function. Initial fMRI analysis was dominated by mass univariate task activation analysis [23], and only in the early 2000s did processing of multivariate patterns of neuronal signals emerge [58]. Multivariate pattern analysis (MVPA) is a broad term describing methods of machine learning that aim to decode neuronal activity as response patterns rather than as isolated brain regions. It demonstrated a distributed representation of high-level visual perception for faces and objects [26]. While mass univariate analysis struggles to reveal distributed effects, machine learning approaches make it possible to capture and model the multivariate phenomenon of brain activity, providing a more complete picture of neural activation.

**Fig. 2:**
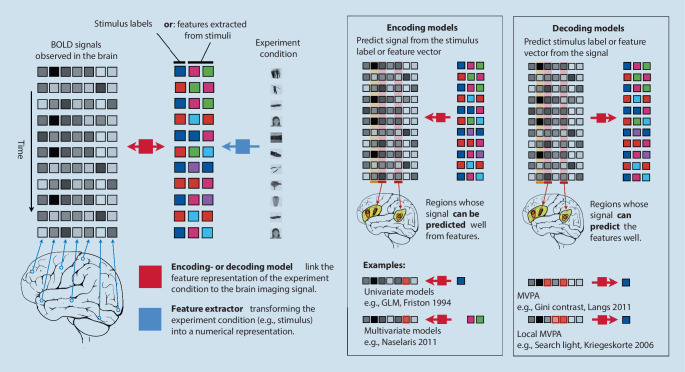
Encoding or decoding models link a feature representation of the experiment condition during functional neuroimaging to the image information. For every time point, image information is represented as a feature vector consisting of the values of each voxel. Correspondingly, the experiment condition is represented by a feature vector, either consisting of labels (house, face, etc.) or features extracted from the condition (e.g., wavelet decomposition of the image, semantic embedding of a word). Encoding models predict image features from the experiment condition features, while decoding models predict in the opposite direction

Machine learning models link the observed neuroimaging information such as the sequential BOLD signal observed for each image voxel of fMRI data to experiment conditions, aiming to identify brain regions whose functional signal is associated with the condition (Fig. 2). *Encoding models* attempt to predict the image signal at each voxel based on the experiment condition. They then test for each individual voxel if its signal can be predicted from experiment conditions such as the class of the visual stimulus. Univariate encoding models such as the GLM [23] are a prominent example, testing each individual voxel independent of the others. If prediction is possible, then GLM treats it as evidence of a significant association or “activation” of this region by the experiment condition. Multivariate encoding models represent the experiment condition with a feature vector instead of a single on/off label. Examples are the representation of words with the help of semantic features to investigate the mapping of semantic concepts across the cortex [59], or the extraction of visual features from images or movies to establish a cortical map of the representations of visual concepts [60, 61]. *Decoding models* predict experiment condition features from the brain imaging data. Typically, a feature selection method is then used to identify sets of features, i.e., voxels, that contribute to a successful prediction of the condition from the imaging data. In contrast to univariate models that test each voxel’s association (e.g., correlation) with the condition independently of others, multivariate models treat the functional imaging data as a pattern possibly consisting of many distributed voxels [62]. Therefore, multivariate models can identify distributed areas, for which individual tests might not identify a relationship, but which taken as a whole actually carry information about the experiment condition. Examples of such approaches are the identification of distributed patterns associated with face processing [26] that spread beyond areas connected in a univariate manner such as the fusiform face area [24]. The so-called searchlight technique proposed an intermediate approach, where instead of individual voxels, the ability of functional patterns observed in localized cortical patches to predict experiment conditions was tested [25]. Ensemble learning approaches such as random forests were used to identify widely distributed patterns associated with visual stimuli. Ensemble learning approaches have a property called the “grouping effect”; they identify all informative voxels, even if they are highly correlated, as opposed to only selecting the single most informative one. Consequently, their feature-scoring methodology, called “Gini importance,” makes it possible to more reliably detect patterns of activation in the form of the so-called Gini contrasts with higher reproducibility [27] than approaches such as support vector machine-based recursive feature elimination [63].

Decoding models have introduced the capability to reconstruct stimuli from observed brain activation. While “mind-reading applications,” such as using fMRI as a lie detector, are not feasible with fMRI [64], remarkable results have been achieved for the decoding of visual stimuli [60] and even dreams [65]. Even for video decoding, a regularized regression approach was used to model dynamic brain activity and was successfully applied to generate similar videos as seen based on the neuronal response patterns in the visual cortex [66]. Similar remarkable progress was made with the decoding of the semantic landscape of natural speech-related brain activity [67], and recent advances in brain decoding have shown that deep learning models can establish a predictor of eye movement from fMRI data [68].

As the number of available data have increased, deep learning models have become relevant in functional neuroimaging. While conventional linear models can be on par with deep learning approaches [69], recent studies have demonstrated that deep learning encodes robust discriminative neuroimaging representations to outperform standard machine learning [70].

### Brain networks and their change during disease

**Fig. 3:**
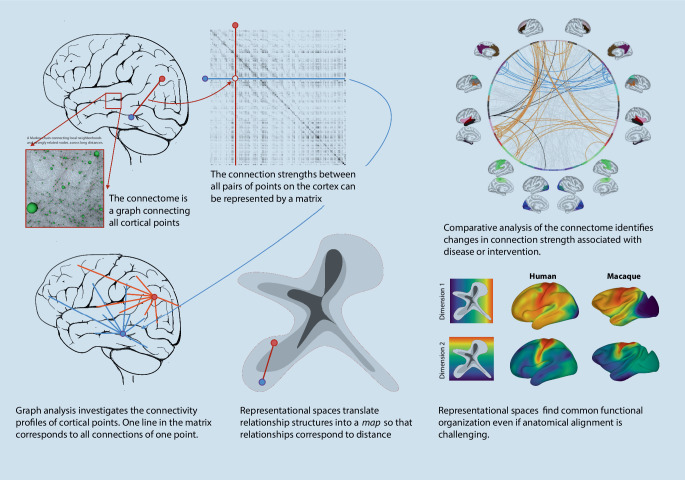
Network analysis investigates the connections among all points in the brain or in the cortex. Connections can be defined by functional MRI signal correlation, or by structural connectivity acquired with diffusion tensor imaging. Connection strengths or presence is represented in a matrix, based on which graph analysis can quantitatively compare networks across cohorts [71]. Representational spaces can capture the connectivity structure and enable its use as a reference when tracking reorganization during disease and treatment, or when comparing the functional layout across species with substantially different anatomy [72]

Neuroimaging is able to capture structural connectivity in the form of nerve fiber bundles with diffusion tensor imaging (DTI) or functional connectivity in the form of correlation among fMRI signals. This enables the analysis of structural or functional brain networks, an area called “connectomics” [73]. Each point on the cortex is viewed as a node in a graph. The connections between pairs of nodes are assigned a measure of connectivity, defined, for instance, by the correlation of fMRI signals observed at the two nodes (Fig. 3). Graph analysis then measures the connectivity properties of individual nodes, with characteristics ranging from the number of connections of a node, the so-called degree, to the role of the nodes in connecting otherwise relatively dissociated networks, the so-called hubness [74]. Statistical analysis of networks [75] has led to insights into the differences of networks across cohorts and the changes during disease progression or during brain development [76]. Representational approaches to render global network structures comparable, and to find components of networks in other networks (see “Machine learning for alignment” section), have led to the ability to track network changes during reorganization in tumor patients [29] or to model the brain network during maturation [77]. Brain network structures are more challenging to process with deep learning methods than image data. Instead of a regular grid structure as in the voxel representation, graphs are largely irregular, and their neighborhood relationships—a critical component of deep artificial network architectures—are heterogeneous. Nevertheless, research has become active in the area of graph convolutional neural networks in neuroimaging [78].

### The relationship between biological and artificial neural systems

Machine learning does not only provide a toolbox for the analysis of neuroimaging data. Methodological advances in fields such as deep learning and artificial neural networks are heavily guided by our understanding of biological networks. The relationship between biological instances and computational implementations of the three core components—objective functions, learning rules, and network architectures—is attracting increasing scientific attention [79]. Deep neural networks have been used as a model to understand the human brain [80], and in the opposite direction, the topology of human neural networks has been used to shape the architecture of artificial neural networks [81]. The quantitative comparison of real biological neural networks and artificial neural networks is challenging, not least because the best computational learning algorithms do not fully correspond to biologically plausible learning mechanisms [82]. Nevertheless, fMRI offers comparative analysis of neural systems, as developed for cross-species analysis [83]. To this end, the dissimilarity of functional activation when processing different visual stimulus categories can be measured in fMRI. At the same time, it can be read out from perceptron activations in artificial neural networks. While the individual activations are not comparable, their dissimilarity structure is, leading to insights into how convolutional neural networks trained to classify objects resemble parts of the human inferior temporal lobe [84]. This has inspired research into the increasingly fine-grained mapping between artificial convolutional neural networks and the visual cortex [85].

### Clinical relevance of machine learning and functional imaging

The brain network architecture is critical for our cognitive capabilities and can be affected by disease [86]. Hence, quantifying and understanding associated changes in the network architecture, or modeling reorganization mechanisms associated with disease progression and recovery, are clinically relevant. Clinical application is challenging, but initial results show promise [87]. Machine learning enables the comparison of networks, the detection of anomalies, and the identification of associations between disease and network alterations up to the establishment of early markers preceding more advanced disease. Deviations in the connectivity structures from a normative model have been found to be predictive of brain tumor recurrence up to 2 months in advance [88]. Alterations in functional connectivity patterns were also predictive of cognitive decline and showed different manifestations in low- and high-grade gliomas [89]. Patients suffering from epilepsy or undergoing epilepsy surgery exhibit specific reorganization patterns of network architecture [71, 90]. Supervised machine learning using functional connectivity data revealed disease correlates not visible in structural imaging, for instance, deep learning models for the identification of characteristics of autism spectrum disorders [91] or a generative autoencoder model to classify autism [92]. An example of the relevance of brain network dynamics is the investigation of patients with schizophrenia [93]. Electroencephalography is a cost-effective and widely available tool in the clinical routine for early diagnosis, and its high temporal resolution can be leveraged by machine learning approaches [94]. Deep convolutional networks utilizing electroencephalography recordings have been successfully applied to detect and classify seizures in patients with epilepsy [95] and to classify attention deficit hyperactivity disorder [96].

## Machine learning for alignment

Establishing reliable correspondence across the brains of individuals and cohorts is essential for group studies and for probing the impact of disease or intervention on an individual’s brain. Therefore, a variety of image registration approaches have been proposed to align individual brains to a common reference frame based on their anatomical properties [97].

### Machine learning for structural image registration

Structural registration methods for the entire brain volume or cortical surface optimize an objective function by deforming one image to match another. Here, machine learning approaches to improve alignment of anatomy have contributed to increasing speed with techniques such as voxel morph [31] for entire volumes, or methods aligning cortical surfaces [98].

### Machine learning for functional alignment

Can we establish alternative bases for registration, if anatomical correspondence is only loosely coupled to functional roles as is the case in the prefrontal cortex [99] or after reorganization due to disease? The anatomy of the brain is highly variable [100] and even after structural alignment a substantial amount of functional variability remains heterogeneously distributed across the cortex [99]. Machine learning offers a means to align individual brains based on their functional imaging data. The individualized functional parcellation of the cortex offered the first way of establishing correspondence independently of the anatomical frame by iterative projection [101] or Bayesian models [102]. Functional response patterns themselves can be mapped to a representational space, where correspondence can be established by so-called hyper-alignment of functional response behavior, even if it occurs at different cortical locations [28]. Finally, the functional connectivity architecture can be represented in an embedding space decoupled from its anatomical anchors, enabling the tracking of reorganization across the cortex in patients with brain tumors [29]. This allows us to investigate shared functional architecture across a large population and has led to insights into the continual hierarchical structure of the cortical processing in the form of so-called functional gradients [103]. The decoupling of anatomical and functional alignment has shown that the link between anatomy and function is not equal across the cortex, but high in primary areas, and comparatively low in association areas or the prefrontal cortex [104]. It has also enabled the comparison of neural architecture across species, since despite cross-anatomical differences there is sufficient similarity in the functional connectivity structure to match and compare across human and macaque cortex [72]. Decoupling anatomy and function has further enabled the study of the different impact of genes and environment on the cortical topography of functional units and their interconnectedness [105]. Recently, it has been shown that functional alignment can improve the generalization of machine learning algorithms to new individuals [106].

## Summary and challenges

Machine learning plays an increasingly important role in the exploitation of neuroimaging data for research and clinical applications. Its capabilities range from the computational segmentation of anatomical structures and the quantification of their properties to the detection and characterization of disease-related findings, such as tumors. In functional imaging, machine learning is able to link distributed activation patterns to cognitive tasks. It is starting to enable the ability to track and model processes such as reorganization, disease progression, or recovery. Unlike many other application fields of machine learning, neuroimaging is itself a source of methodological advancements in areas such as deep learning. There, the comparison of artificial network architectures and learning algorithms with biological mechanisms yields anchors for novel methodology and fosters insights into the working of the central nervous system.

The current clinical relevance of machine learning is based on its ability to detect, quantify, track, and compare anatomy and disease-related patterns. Some of the most promising challenges facing the field currently comprise three central directions. First, for the linking of phenotypic data observed in neuroimaging to underlying biological mechanisms, machine learning methodology can bridge the gap between representing imaging data and other molecular markers of processes. Second, embedding methods offer the ability to go beyond the anatomical reference frame when studying brain architecture and its change during disease, treatment response, and recovery. As the link between anatomy and function loses critical importance as a basis for analysis, we gain the ability to study their intricate relationship and its variability. Finally, we need to improve our understanding of differentiating the vast natural variability of the brain’s anatomy and function and the often subtle deviations associated with disease.

## Practical conclusion

- Machine learning techniques can detect and segment anatomical structures and findings associated with disease to support diagnosis and to provide quantitative characterizations.
- Encoding and decoding models identify brain areas whose multivariate functional activity is associated with specific cognitive tasks.
- Graph theoretical methods can analyze and compare brain networks in individuals and across cohorts.
- Representational models can uncouple analysis of function and structure and leverage the connectivity structure to establish correspondences when anatomy is affected by disease.

## Declarations

For this article no studies with human participants or animals were performed by any of the authors. All studies mentioned were in accordance with the ethical standards indicated in each case.

The supplement containing this article is not sponsored by industry.
